# Low quantity and quality of anti-spike humoral response is linked to CD4 T-cell apoptosis in COVID-19 patients

**DOI:** 10.1038/s41419-022-05190-0

**Published:** 2022-08-27

**Authors:** Sonia André, Marne Azarias da Silva, Morgane Picard, Aurélie Alleaume-Buteau, Lucy Kundura, Renaud Cezar, Calaiselvy Soudaramourty, Santa Cruz André, Ana Mendes-Frias, Alexandre Carvalho, Carlos Capela, Jorge Pedrosa, António Gil Castro, Paul Loubet, Albert Sotto, Laurent Muller, Jean-Yves Lefrant, Claire Roger, Pierre-Géraud Claret, Sandra Duvnjak, Tu-Anh Tran, Ouafa Zghidi-Abouzid, Pierre Nioche, Ricardo Silvestre, Pierre Corbeau, Fabrizio Mammano, Jérôme Estaquier

**Affiliations:** 1 Université Paris Cité, INSERM U1124, F-75006 Paris, France; 2 Structural and Molecular Analysis Platform, BioMedTech Facilities INSERM US36-CNRS UMS2009, Université Paris Cité, Paris, France; 3 Laboratoire d’Immunologie, CHU de Nîmes, Nîmes, France; 4 Life and Health Sciences Research Institute (ICVS), School of Health Sciences, University of Minho, Braga, Portugal; 5 ICVS/3B’s—PT Government Associate Laboratory, Braga/Guimarães, Portugal; 6 Department of Internal Medicine, Hospital of Braga, Braga, Portugal; 7 Clinical Academic Center-Braga, Braga, Portugal; 8 Service des Maladies Infectieuses et Tropicales, CHU de Nîmes, Nîmes, France; 9 Service de Réanimation Chirugicale, CHU de Nîmes, Nîmes, France; 10 Urgences Médico-Chirugicales Hospitalisation, CHU de Nîmes, Nîmes, France; 11 Service de Gérontologie et Prévention du Vieillissement, CHU de Nîmes, Nîmes, France; 12 Service de Pédiatrie, CHU de Nîmes, Nîmes, France; 13 CHU de Québec—Université Laval Research Center, Québec City, QC Canada; 14 Institut de Génétique Humaine UMR9002 CNRS-Université de Montpellier, Montpellier, France; 15 INSERM U1259 MAVIVH, Université de Tours, Tours, France

**Keywords:** Infectious diseases, Infection

## Abstract

In addition to an inflammatory reaction, Severe Acute Respiratory Syndrome Coronavirus 2 (SARS-CoV-2)-infected patients present lymphopenia, which we recently reported as being related to abnormal programmed cell death. As an efficient humoral response requires CD4 T-cell help, we hypothesized that the propensity of CD4 T cells to die may impact the quantity and quality of the humoral response in acutely infected individuals. In addition to specific immunoglobulins (Ig)A, IgM, and IgG against SARS-CoV-2 nucleocapsid (N), membrane (M), and spike (S1) proteins, we assessed the quality of IgG response by measuring the avidity index. Because the S protein represents the main target for neutralization and antibody-dependent cellular cytotoxicity responses, we also analyzed anti-S-specific IgG using S-transfected cells (S-Flow). Our results demonstrated that most COVID-19 patients have a predominant IgA anti-N humoral response during the early phase of infection. This specific humoral response preceded the anti-S1 in time and magnitude. The avidity index of anti-S1 IgG was low in acutely infected individuals compared to convalescent patients. We showed that the percentage of apoptotic CD4 T cells is inversely correlated with the levels of specific IgG antibodies. These lower levels were also correlated positively with plasma levels of CXCL10, a marker of disease severity, and soluble Fas ligand that contributes to T-cell death. Finally, we found lower S-Flow responses in patients with higher CD4 T-cell apoptosis. Altogether, these results demonstrate that individuals with high levels of CD4 T-cell apoptosis and CXCL10 have a poor ability to build an efficient anti-S response. Consequently, preventing CD4 T-cell death might be a strategy for improving humoral response during the acute phase, thereby reducing COVID-19 pathogenicity.

## Introduction

Over the last two decades, several highly pathogenic coronaviruses have successively emerged, including SARS-CoV-1, MERS-CoV, and SARS-CoV-2. Patients with SARS-CoV-2 infection develop mild to severe respiratory illness named coronavirus disease 2019 (COVID-19), which can be lethal. Dysregulated inflammatory immune response, cell damage, or the pro-coagulant state induced by SARS-CoV-2 infection are among the factors that may contribute to disease severity and outcome [1].

SARS-CoV-2 encodes for several antigens, including the Membrane (M), Spike (S), and Nucleocapsid protein (N) that induce specific Immunoglobulins (Ig). However, controversial results have been reported regarding the humoral response in individuals infected by SARS-CoV-2. Although several reports have shown an early humoral response directed against S and/or the receptor binding domain (RBD) with similar IgA, IgG, and IgM dynamics [2–4], others have reported that IgA dominates the early antibody response to SARS-CoV-2 [5]. In severe forms of the disease, a delay in the development of anti-S IgM and IgG has been noted compared to mild disease [6, 7], whereas others have reported a compromised humoral response [8–10]. This is also mirrored by the more robust development of memory B cells during moderate COVID-19 forms compared with severe forms of COVID-19 disease [11]. Convalescent patients display sustained production of the neutralizing IgG antibody for several months [12–14]. In addition, convalescent patients who display the highest prevalence of neutralizing antibodies have been reported to have the highest anti-S IgG avidity [15, 16]. However, little attention has generally been paid to measuring the avidity of antibodies during SARS-CoV-2 infection that determines the quality and strength of an antibody-antigen complex [17, 18] when compared to other measurements (ELISA, chemiluminescence, and flow cytometry).

CD4 T cells are essential for sustaining germinal center (GC) formation and B-cell differentiation leading to an isotype switch and immunoglobulin (Ig) maturation, two features of T-cell-dependent humoral response [19–21]. Several groups have reported a clear association between the extent of T-cell immunity and humoral response in convalescent individuals [22–24]. However, lymphopenia is observed in two-thirds of COVID-19 patients [25], associated with a defect in Th1-cell-mediated immunity during the acute phase of infection [22]. Defective GC formation associated with CD4 T-cell depletion in the lymph nodes of severe COVID-19 patients has been also reported [26]. Furthermore, we demonstrated that T cells from COVID-19 patients were more prone to die by apoptosis correlating with this lymphopenia [27]. Thus, we hypothesized that the propensity for CD4 T cells to die in the early phase of infection might be associated with weaker humoral responses against SARS-CoV-2.

We hereby evaluated the specificity and quality of COVID-19 humoral response by accurately assessing the IgG, IgA, and IgM responses using secondary antibodies that were only directed against the heavy chain of these Igs (gamma, alpha, and mu, respectively)—not the light chains—thus increasing the specificity of our assays. Our results highlight that viral-specific IgA predominates during the early phase of infection, in which N represents a strong antigenic candidate for monitoring early SARS-CoV-2 infection. The IgG response against S was delayed, displayed the lowest avidity in more severely affected individuals, and was even absent in nonsurvivors. This early humoral response was negatively correlated with the age of COVID-19 patients and plasma CXCL10 levels. Furthermore, we demonstrate that the levels of CD4 T-cell death and soluble Fas ligand (sFasL) correlate with weaker IgG responses in COVID-19 individuals. This correlates with the hypothesis that early T-cell death might contribute to the pathogenesis of COVID-19. Therefore, these results may provide valuable insights for understanding the dynamics of the immune response and pave the way for new advances in clinical diagnosis and predicting the outcome of COVID-19 infection.

## Results

### Low levels of the humoral response against the SARS-COV-2 spike protein in COVID-19 patients upon admission

**Fig. 1:**
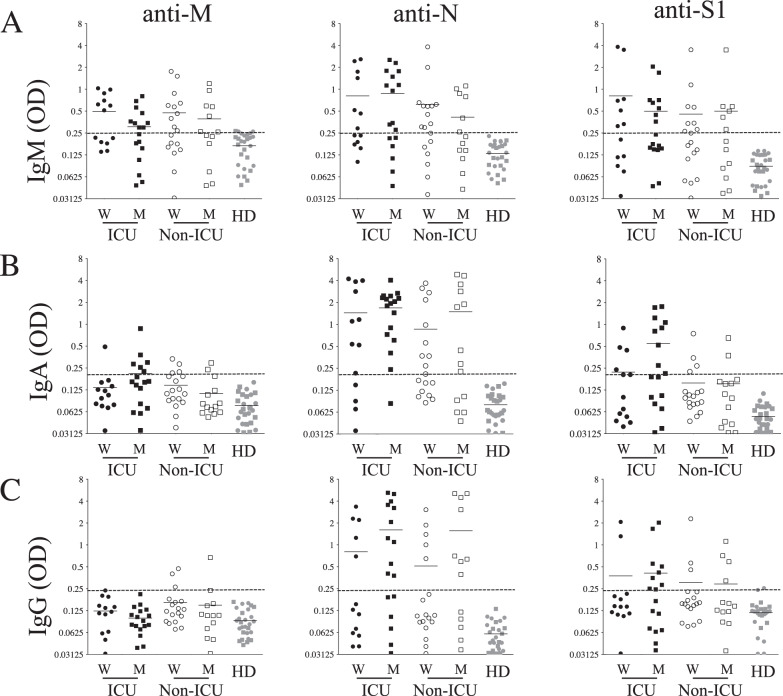
Humoral response in SARS-CoV-2-infected individuals. Plasma was diluted to 1/800, 1/400, and 1/400 for **A** IgM, **B** IgA, and **C** IgG, respectively, and tested against membrane (M), nucleocapsid (N), and spike (S1) proteins. Each dot represents an individual, wherein black symbols are ICU patients, white symbols are non-ICU patients and gray symbols represent healthy donors (HD). Circles are females (ICU_W_ and non-ICU_W_) and squares are males (ICU_M_ and non-ICU_M_). OD optical density is shown. Dashed lines represent antibody specificity as defined in the supplementary figures.

The first cohort used for this study included 62 patients hospitalized in the intensive care units (ICU, *n* = 30) and non-ICU (*n* = 31). Their demographic and clinical characteristics are detailed in Table 1 and compared with 31 age- and gender-matched healthy donors (HDs). Among the hospitalized patients, six did not survive. We assessed the humoral response by analyzing plasma samples from COVID-19 individuals obtained on the day of hospitalization and from HDs. Specific antibodies against a SARS-COV-2 S1 subunit of the spike protein (S1), M, and N proteins were titrated by serial dilution using highly specific secondary antibodies against IgM, IgA, and IgG (Supplementary Figs. 1–3) Indeed, antibody class switch recombination (CSR) may be indicative of CD4 T-cell help [19, 20, 28]. The optical densities shown in Fig. 1 are the values obtained at dilutions of 1/800, 1/400, and 1/400 for specific IgM, IgA, and IgG responses, respectively.

**Table 1:** Clinical characterization of ICU and non-ICU individuals.

Parameter	*N* (% or range) Non-ICU versus ICU
Gender, *n* (%)	
Female	18 (56) versus 13 (43)
Male	13 (44) versus 17 (57)
Age, years (median)	70 (29–96) versus 71 (43–95)
Symptoms, *n* (%)	
Cough at admission	6 (18) versus 11 (36)
Dyspnea at admission	7 (22) versus 16 (53)
Fever at admission	6 (18) versus 25 (83)
Reasons for admission, *n* (%)	
Hypoxemia	16 (51) versus 30 (100)
Gastrointestinal disorders	7 (22) versus 0 (0)
Other medical conditions	6 (18) versus 0 (0)
Treatment, *n* (%)	
Patients supported by oxygenotherapy	26 (83), non-ICU
Patients supported by invasive ventilation	30 (100), ICU
Hydroxychloroquine (monotherapy)	0
Hydroxychloroquine + azithromycin	3
Outcome, *n* (%)	
Death (ICU)	6 (20)

**Fig. 2:**
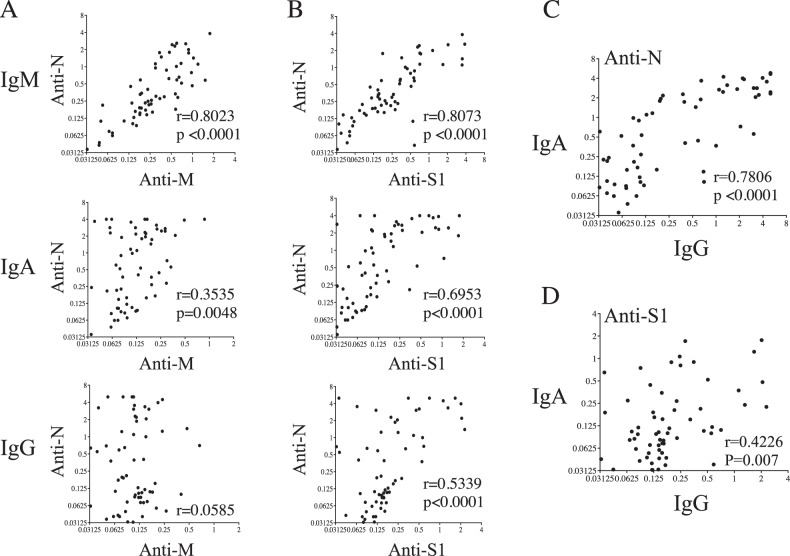
Correlations between humoral responses. IgM, IgA, and IgG responses against **A** N versus M and **B** N versus S. Top: IgM antibodies; Middle, IgA antibodies; and Bottom, IgG antibodies. Correlations between specific IgA and IgG antibodies against **C** N and **D** S1 antigens. Values are derived from Fig. 1. OD optical density. Each dot represents an individual. Correlations were assessed using the Spearman test and r is indicated.

Our results demonstrated that half the patients hospitalized for COVID-19 had developed IgM specifically directed against M (16/30 of ICU and 17/31 of non-ICU were responders), N (18/30 of ICU and 17/31 of non-ICU), and S1 antigens (15/30 of ICU and 14/31 of non-ICU) when compared to HDs (Fig. 1A). Thus, 57% of COVID-19 patients had IgM antibodies directed against N, whereas 54% and 48% developed anti-M and anti-S1 IgM, respectively. No statistically significant differences were observed among anti-S1, -M, or -N IgM between ICU and non-ICU COVID-19 patients or between female and male patients (Fig. 1A). By contrast, in a separate group of 20 convalescent patients’ samples, 6 months after acute infection, specific IgM were only rarely detected (Supplementary Fig. 4A). Since the age of patients may influence humoral response [29], Ig response was reanalyzed separating patients into age groups (above or below 70 years). Interestingly, a significant decrease was observed in older ICU patients in whom IgM responses directed against N and S1 were lower compared to younger ICU individuals (Supplementary Fig. 5A, B). However, no age-related difference was observed for non-ICU patients. A strong positive correlation was observed between anti-M and anti-N (r = 0.8023, *p* < 0.0001) and between anti-S1 and anti-N IgM responses (r = 0.8073, *p* < 0.0001) (Fig. 2A, B, top panels) demonstrating similar IgM responses against these three SARS-CoV-2 proteins in COVID-19 patients.

We then assessed the IgA response against M (7/30 of ICU and 5/31 of non-ICU were responders), N (25/30 of ICU and 19/31 of non-ICU), and S1 antigens (12/30 of ICU and 6/31 of non-ICU) (Fig. 1B). Interestingly, while 72% of COVID-19 patients had IgA anti-N antibodies, only 19% had IgA anti-M and 29% anti-S1. As observed for IgM, specific IgA was rarely detected in convalescent individuals (Supplementary Fig. 4A). A positive correlation was observed between anti-N and anti-S1 IgA responses (Fig. 2B, middle panel, r = 0.6953, *p* < 0.0001). Regarding patients’ gender, the frequencies of anti-IgA were similar for ICU and non-ICU patients. Furthermore, although most older ICU individuals (>70 years old) had anti-N IgA (65%), fewer than 10% of them had IgA against S1 (Supplementary Fig. 5A, B). In the ICU patients, the levels of IgA antibodies were higher in individuals under 70 years old. In contrast to ICU patients, the age of non-ICU individuals had no impact on IgA response against N or S1 (Supplementary Fig. 5A and B). Thus, IgA humoral response against N represents a predominant humoral response which might well be of interest as an early immune diagnostic marker, independent of patients’ gender and less impacted by age than anti-S1 IgA antibodies.

Finally, we analyzed specific IgG responses against M (1/30 of ICU and 5/31 of non-ICU were responders), N (16/30 of ICU and 13/31 of non-ICU), and S1 antigens (11/30 of ICU and 7/31 of non-ICU) (Fig. 1C). The frequency of IgG responders against N (47%) was higher than that observed for S1 (29%, χ2, *p* = 0.04) and M antigens (10%, χ2, <0.0001). This was markedly different from the situation observed in convalescent individuals in whom anti-N and anti-S1 IgG predominated (Supplementary Fig. 4A). Although no statistically significant differences were found between acutely infected ICU and non-ICU patients (Fig. 1C), the frequency of male responders against N (19/30) was significantly higher compared to the frequency of female responders (10/21, χ2, *p* = 0.015). A positive correlation was observed for the IgG response between N and S1 antigens (Fig. 2B, bottom panels). Similar to the IgM and IgA response, the response of IgG against N and S1 was lower in older patients (>70 years) than in younger ICU patients (<70 years) (Supplementary Fig. 5A, B). A positive correlation was observed when plotting the IgA versus IgG anti-N humoral responses (r = 0.7806, *p* < 0.0001; Fig. 2C), whereas this correlation was lower with anti-S1 humoral response (Fig. 2D).

Therefore, although the IgM responses were equivalent for N, S1, and M (56%, 50%, and 49% of responders, respectively), the frequencies of specific IgG (47%) and IgA (72%) against N were higher than for specific IgG (29%) and IgA (29%) anti-S1. Altogether, our results demonstrated that COVID-19 patients had low levels of IgG and IgA response against S1 upon admission, suggesting that antibody CSR of anti-S1 humoral response is impaired.

### Humoral response against the SARS-COV-2 spike protein is delayed in time and magnitude

**Fig. 3:**
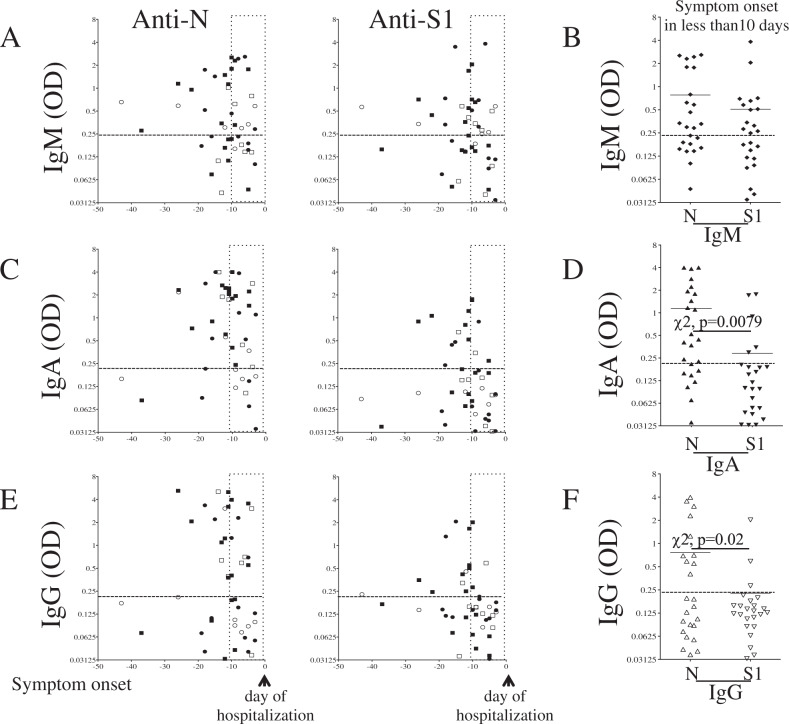
Symptom onset and humoral response. **A, B** IgM, **C, D** IgA, and **E, F** IgG responses against nucleocapsid (N) and spike (S1) proteins. Values are derived from Fig. 1. Each dot represents an individual. Black symbols are ICU patients, white symbols are non-ICU patients. Circles are females and squares are males. **B** IgM (diamonds), **D** IgA (filled symbols), and **F** IgG (blank symbols) responses in individuals for whom symptom onsets were less than 10 days. Horizontal dashed line indicates the limit of specificity and the vertical dashed line shows 10 days before the day of hospitalization. A chi-squared test (χ2 test) was used to compare the frequencies of responders.

Having observed a lower S1 humoral response, we assessed whether the delay between symptoms onset and hospitalization was associated with the establishment of different Ig responses. The onset of symptoms in individuals in whom anti-N Ig were detected (responders) was −13.4 ± 1.9 days for IgM (Fig. 3A), −11.5 ± 5.6 days for IgA (Fig. 3C), and −14.5 ± 5.6 days for IgG (Fig. 3E), and for anti-S1 the onset of symptoms was −13.1 ± 1.8 for IgM, (Fig. 3A) −11.2 ± 5.3 days for IgA (Fig. 3C), and −12 ± 3.5 days for IgG (Fig. 3E). By analyzing the humoral response for patients whose symptoms appeared less than 10 days before hospitalization, the frequencies of IgM against N (14/24) and S (13/24) were similar (Fig. 3B). However, we observed that anti-N responses predominated for both IgG and IgA. Thus, 14/24 individuals were IgA responders for N, whereas only 5/24 were responders for S1 (χ2, *p* = 0.0002) (Fig. 3D). Similarly, 10/24 individuals were IgG responders for N, whereas only 3/24 were responders for S (χ2, *p* = 0.0077) (Fig. 3F). Thus, the humoral response against N developed earlier when compared to anti-S response based on symptom onset.

**Fig. 4:**
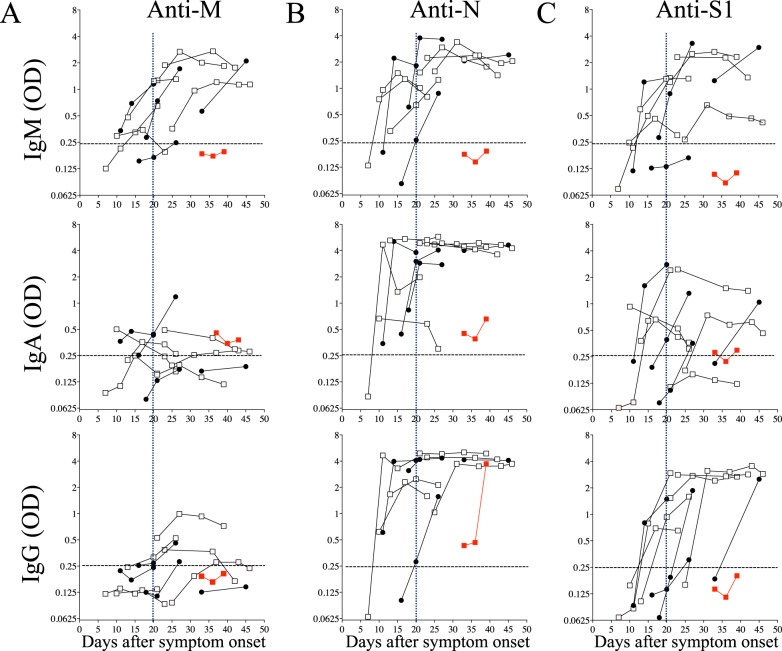
Humoral response after symptom onset. Plasma after symptom onset was diluted as described in Fig. 1 and tested against **A** membrane (M), **B** nucleocapsid (N), and **C** spike (S1) proteins. Each dot represents an individual. Blank squares and filled circles are males and females, respectively. The red dot represents the nonsurviving individual. The horizontal dashed lines represent the antibody specificity and the vertical dashed lines represent 20 days from symptom onset.

To extend this analysis, we assessed the dynamics of the humoral responses in the second group of patients who had been hospitalized and followed up at different time points after their hospitalization (Table 2). The duration of hospitalization in this COVID-19 cohort was 22 ± 2.2 days. This group consisted of 4 women and 7 men, among whom one man did not survive. Consistent with our previous data, we found that the humoral response directed against the M antigen was mainly an IgM response (Fig. 4A). Four individuals developed anti-M IgA and IgG responses over time but at considerably low levels (Fig. 4A). By contrast, the N antigen was extremely potent in generating, not only IgM but also specific IgA and IgG (Fig. 4B). Among these patients, 91% displayed specific IgA and IgG antibodies 20 days after symptom onset.

**Table 2:** Clinical characterization of patients monitored during the acute phase of hospitalization.

Parameter	*N* (% or range)
Gender, *n* (%)	
Female	4 (36)
Male	7 (64)
Age, years (range)	70 (44–89)
Underlying diseases, *n* (%)	
Autoimmune Disease	1 (9)
Cancer history	2 (18)
Hypertension	6 (55)
Diabetes	2 (18)
Chronic Obstructive Pulmonary Disease	2 (18)
Other respiratory disease	1 (9)
Chronic Kidney Disease	1 (9)
Symptoms, *n* (%)	
Days of symptoms before admission	9 (4–22)
Cough at admission	8 (73)
Dyspnea at admission	8 (73)
Fever at admission	8 (73)
Reasons for admission, *n* (%)	
Hypoxemia	8 (73)
Gastrointestinal disorders	1 (9)
Other medical conditions	2 (18)
Treatment, *n* (%)	
Patients supported by noninvasive ventilation at any point	4 (36)
Patients supported by invasive ventilation at any point	2 (18)
Hydroxychloroquine (monotherapy)	2 (18)
Hydroxychloroquine + azithromycin	4 (36)
Any of the above + corticosteroids	4 (36)
Corticosteroids (monotherapy)	1 (9)
Outcome, *n* (%)	
Deaths	1 (9)
Cured	10 (91)

**Fig. 5:**
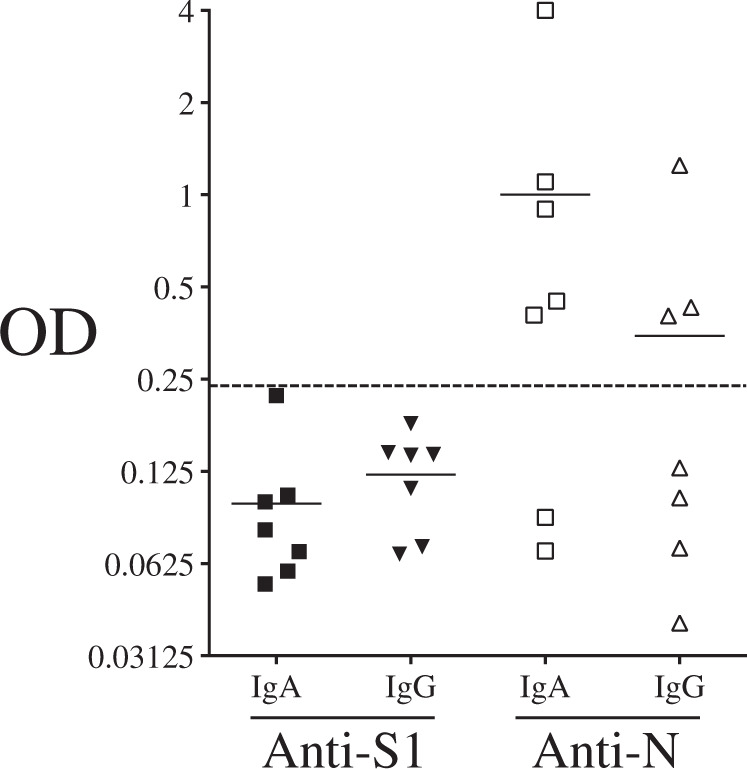
Humoral responses in nonsurvivors and IgA and IgG response against S1 and N antigens. IgA (square) and IgG (triangle) response against the nucleocapsid (N, blank symbol), the spike (S1, filled symbol) protein. Each dot represents an individual. Dashed lines represent antibody specificity (responders).

The anti-S1 IgA response was delayed and with less intensity than the anti-N response (Fig. 4C, middle). A similar observation was made for the anti-S1 and anti-N IgG responses (Fig. 4C, bottom). Only half the patients had specific anti-S1 IgG compared to the 81.8% who developed anti-N IgG on Day 20 after symptom onset. However, the extent of anti-S1 IgG was higher when compared to IgA (Fig. 4C, middle versus bottom). Because we observed a lower humoral response in the individual who did not survive (Fig. 4, in red), we extended the analysis to non-survivor patients in both cohorts (Fig. 5). Although 5 patients out of 7 displayed specific IgA and 3 out of 7 specific IgG against N, none of them developed specific anti-S1 humoral responses.

Altogether, our results underline the value of the IgA response against the viral N protein for early diagnosis. The data also demonstrated a considerable delay in producing a significant humoral response against the S1 protein compared with N, suggesting that the production of anti-S1 antibodies has additional constraints.

### Low avidity of antibodies against the SARS-COV-2 spike protein in COVID-19 patients upon admission

**Fig. 6:**
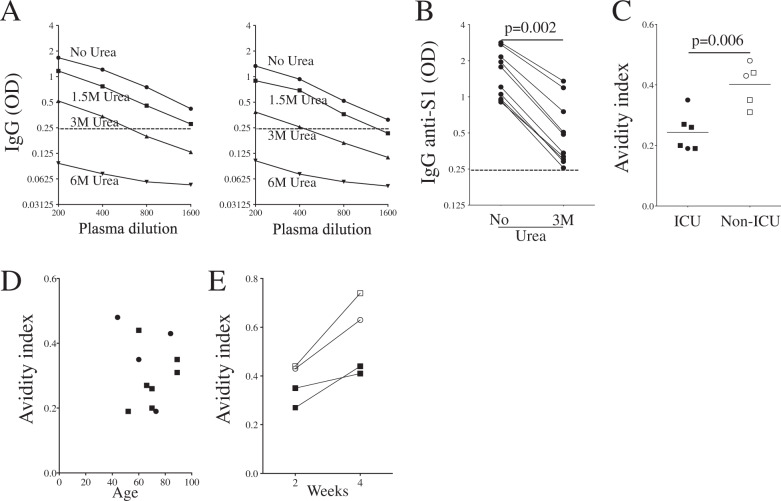
Avidity. **A** Specific anti-S1 IgG detected from the diluted plasma of two ICU individuals in the absence (No Urea) or presence of increasing concentrations of Urea (1.5, 3, and 6 M) are shown. **B** Specific S1 IgG in the absence or presence of urea at 3 M. Plasma was diluted to 1/400. A nonparametric paired Mann–Whitney test was used for comparison. **C** The avidity index (ratio: OD with 3 M of urea/OD without urea) was measured in ICU and non-ICU individuals from panel **B**. A nonparametric Mann–Whitney test was used to compare ICU and non-ICU patients. **D** Dot-plot showing the avidity index against the age of the patients (Circles are females and squares are males). Each dot represents an individual. **E** In four individuals (ICU, filled symbol and non-ICU, blank symbol) the avidity index was measured at 2 and 4 weeks after symptom onset as shown in Fig. 5.

CD4 T cells are not only essential for antibody CSR but also for Ig maturation and affinity, which is associated with neutralizing activity [21]. Thus, measuring the avidity reflects the Ig maturation process and, in turn, the help provided by CD4 T cells [15, 16]. By adding a denaturing urea treatment, weak interactions between antibody and antigen are lost. In previous reports [15, 30], a denaturing solution higher than 5 M of urea was used, which was probably too strong to monitor differences between patients showing low levels of avidity index (AI) in the early phase of infection (AI was <0.2). We treated COVID-19 patients’ plasma with increasing urea-based denaturing solution (1.5, 3, and 6 M) (Fig. 6A). The highest concentration of urea (6 M) completely inhibited antigen-antibody binding in ICU patients, whereas the lower concentration (1.5 M) was not strong enough to impair the binding interaction. Therefore, 3 M was used to measure AI. The binding of specific anti-S1 IgG was significantly reduced in the presence of 3 M urea (Fig. 6B). Patients included were those who displayed a magnitude of anti-S1 IgG higher than 0.6 OD at 1/400 dilution to prevent a bias in the AI calculation. Non-ICU patients displayed a statistically significant higher AI compared to ICU patients (Fig. 6C). Age or gender did not correlate with the AI (Fig. 6D) in these 11 patients. The AI values increased in Week 4 compared to Week 2 and remained higher in non-ICU patients than in ICU patients (Fig. 6E). Thus in contrast with the acute phase in which the avidity of the antibodies directed against S1 was low (AI < 0.4) (Fig. 6C) AI increased, reaching high levels (>0.7) in convalescent individuals (Supplementary Fig. 4B).

Altogether, our results indicate that specific IgG antibodies against the S1 protein have low avidity in ICU individuals, which may compromise SARS-CoV-2 immune control leading to more severe disease.

### The level of CXCL10 is associated with a lower magnitude of humoral response in COVID-19 patients upon admission

**Fig. 7:**
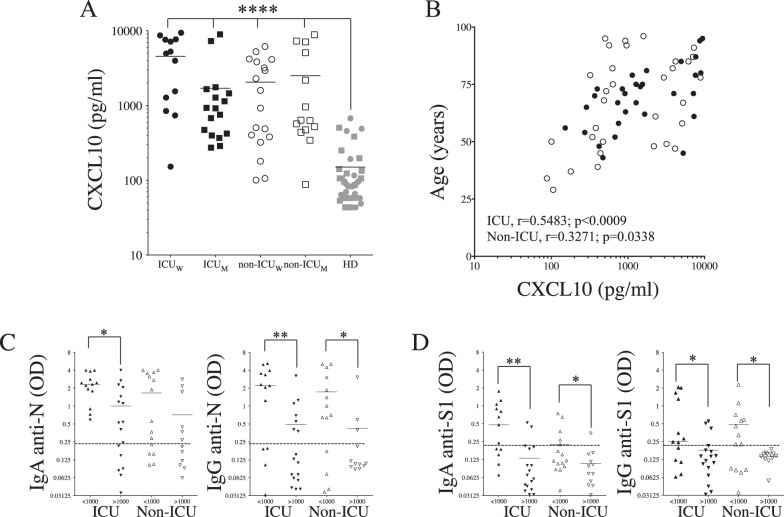
Relationship between levels of CXCL10 and humoral response. **A** Levels of CXCL10. Each dot represents an individual. Back symbols are ICU, white symbols are non-ICU, and gray HDs. Circles are females (ICU_W_ and non-ICU_W_) and squares are males (ICU_M_ and non-ICU_M_). **B** Correlation between age and levels of CXCL10. Correlations were assessed using the Spearman test and r is indicated. IgA and IgG responses against N **C** and S1 **D** of ICU and Non-ICU with higher (>1000 pg/mL) and lower (<1000 pg/ml) of CXCL10. A nonparametric Mann–Whitney test was used for comparison (*p* values: *, <0.05; **, <0.01; ***, <0.001).

Recently, a negative correlation between humoral response and the extent of inflammation has been proposed [31]. We, and others, have found that, among several interleukins and chemokines, CXCL10 (interferon-γ-inducible protein-10 or IP-10), a well-known chemokine [32, 33] that recruits immune cells expressing CXCR3 [34] and contributes to lung inflammation in several viral diseases [35, 36], correlates with the extent of SARS-CoV-2 viral replication in the tissues and is a strong marker of COVID-19 disease severity [27, 37, 38]. Thus, we assessed the levels of inflammatory CXCL10 in the plasma of both ICU and non-ICU individuals. We found higher plasma levels of CXCL10 in COVID-19 patients compared to HDs (Fig. 7A). More importantly, our results highlighted that levels of CXCL10 correlated positively with the age of COVID-19 patients requiring ICU admission (r = 0.5483, *p* < 0.0009) (Fig. 7B). We observed a negative correlation between levels of CXCL10 in the plasma and anti-N and anti-S1 IgA humoral response as well as for anti-N IgG in ICU patients (Supplementary Fig. 6). Having observed that patients with high levels of CXCL10 (>1000 pg/ml) displayed lower levels of humoral response, we decided to reanalyze humoral response in patients based on this threshold (higher or lower than 1000 pg/ml). Our data indicate that individuals with higher levels of CXCL10 have significantly lower levels of specific antibodies (Fig. 7C, D).

Altogether, these results indicate that the humoral response is inversely correlated with the level of plasma CXCL10, a biomarker associated with disease severity and age of COVID-19 patients.

### CD4 T-cell death and sFasL are associated with lower humoral response in COVID-19 patients upon admission

**Fig. 8:**
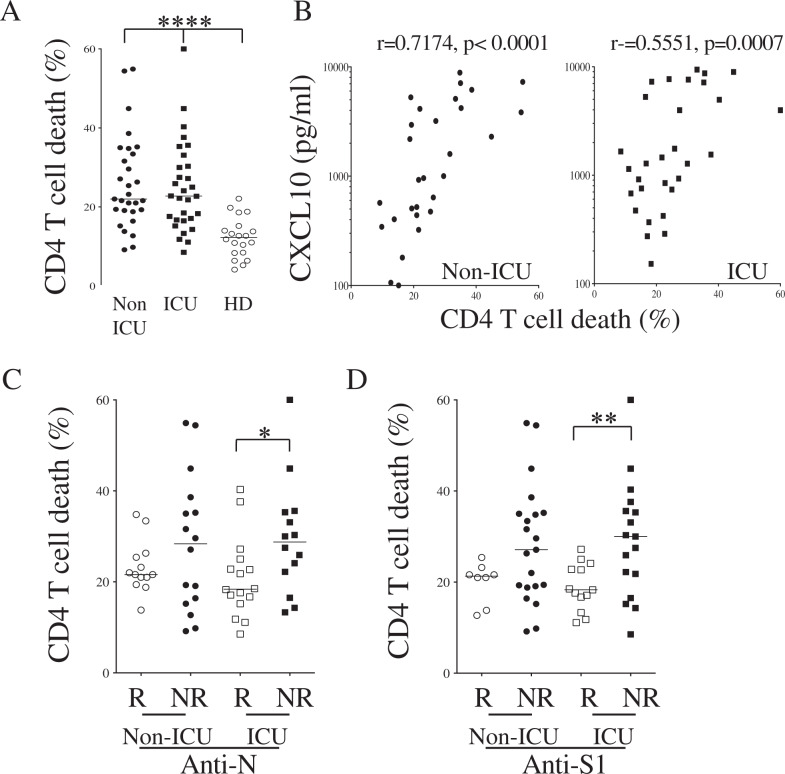
CD4 T-cell death and levels of IgG against N and S1 SARS-CoV-2 antigens. **A** CD4 T-cell death was quantified by flow cytometry using a fluorescent caspase substrate. **B** Dot plots show the correlation between the percentage of CD4 T-cell death and the levels of CXCL10 in both non-ICU (circle) and ICU individuals (square). **C, D** Levels of CD4 T-cell death in either anti-N (**C**) or anti-S1 **D** responders (R, blank symbols) versus nonresponders (NR, close symbols) of both non-ICU (circle) and ICU individuals (square). Statistical analysis was performed using a Mann–Whitney *U* test (*p < 0.05; **p < 0.01; ****p < 0.0001). Correlations were assessed using the Spearman test and r and p are indicated.

Although CD4 T helper cells are essential for B-cell maturation, germinal center (GC) formation and Ig affinity maturation [19, 20, 28], we recently reported that CD4 T cells from COVID-19 individuals were more prone to die by apoptosis, which is prevented by using a caspase inhibitor, not by using pyroptosis or necroptosis inhibitors [27]. We thus assessed the level of CD4 T-cell death by detecting caspase activity using flow cytometry after ex vivo culture and found that COVID-19 patients’ CD4 T cells were more prone to die than those of healthy donors (HDs) (Fig. 8), consistent with our recent report [27]. Specifically, the percentages of apoptotic CD4 T cells were significantly higher, both in ICU (mean ± SD, 24.9 ± 11.3%) and non-ICU patients (26.1 ± 11.8%), compared to HDs (12.2 ± 4.9%) (Fig. 8A). Thus, more than 60% of SARS-CoV-2-infected individuals, including both non-ICU and ICU patients, displayed higher levels of CD4 T-cell death (>20%) than observed with HDs. Our results also indicate a positive correlation between the extent of CD4 T-cell death and the level of CXCL10, both in non-ICU and ICU individuals (Fig. 8B). Our results revealed that, in the group of ICU patients, the extent of cell death was significantly higher in patients who developed neither anti-N nor anti-S1 IgG (nonresponders) compared to patients in this same group who did develop IgG responses (responders) against the antigens tested (Fig. 8C, D). A similar pattern was observed in non-ICU individuals, with over 60% of them showing higher levels of CD4 T-cell death in nonresponders than responders, although these differences did not reach statistical significance (Fig. 8C, D).

**Fig. 9:**
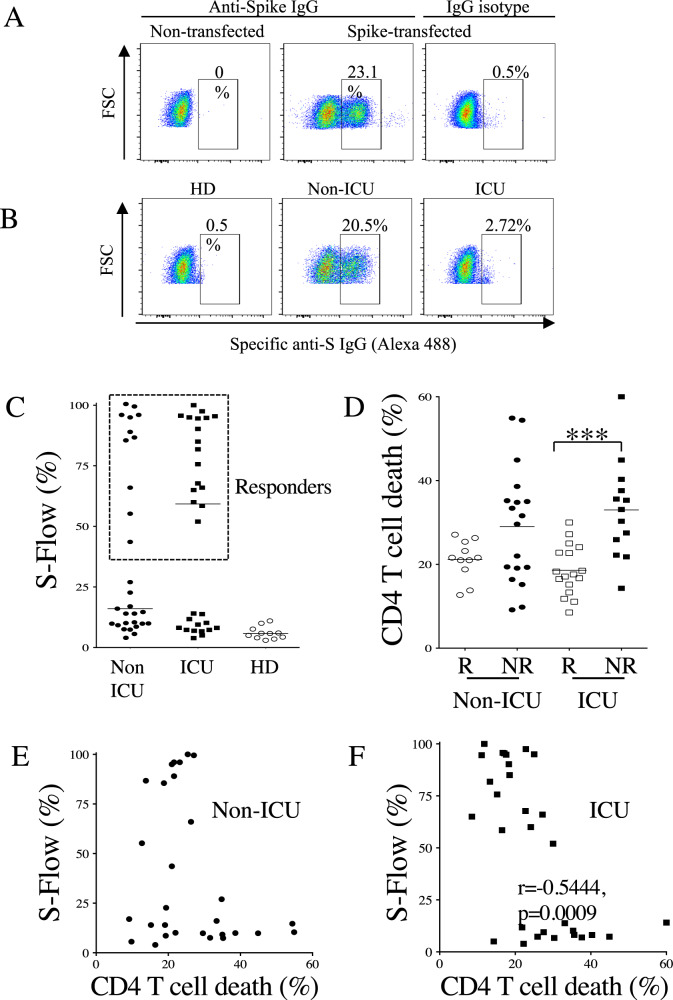
CD4 T-cell death and levels of S-Flow IgG. **A** Cells transfected with the spike gene were used to quantify specific IgG antibodies by flow cytometry (S-flow). As a control to cell transfection, a spike monoclonal antibody was used (anti-spike). **B** Plasmas from a healthy donor (HD), a non-ICU and an ICU were diluted at 1/300. Specific antibodies were detected with Alexa 488-labeled secondary antibodies. The percentages are shown. **C** Percentages of S-expressing cells are indicated for HD, non-ICU, and ICU individuals. Each dot represents one individual. The results are normalized using (anti-spike mAb; % mAb anti-spike − % of Ig isotype) / (% IgG in the plasma − % of Ig isotype)*100. The dashed line separates responders from nonresponders. **D** Dot plots show the percentage of CD4 T-cell death in either S-flow responders (R, blank symbols) versus nonresponders (NR, close symbols) of both non-ICU (circle) and ICU individuals (square). Statistical analysis was performed using a Mann-–Whitney *U* test (***p < 0.001). **E, F** Correlations between S-Flow and CD4 T-cell death in non-ICU (**E**) and ICU (**F**). Each dot represents one individual. Correlations were assessed using the Spearman test and r and p are indicated.

We also assessed the impact of CD4 T-cell death on the capacity of IgG to recognize the S antigen expressed on the cell surface of transfected cells (S-Flow), which correlates with neutralization efficiency [39] (Fig. 9A, B). At a dilution of 1/300, we detected 28/59 responders (non-ICU, 11/29 and ICU, 17/30) (Fig. 9C). By ranking IgG responders and nonresponders, we found that nonresponding ICU individuals had higher levels of CD4 T-cell death than responders (Fig. 9D). In non-ICU patients, although this difference was not significant, over 55% of nonresponders had higher levels of T-cell death than responders. By plotting the extent of CD4 T-cell death against S-Flow responses in non-ICU (Fig. 9E) and ICU individuals (Fig. 9F), a negative correlation was observed for ICU patients.

**Fig. 10:**
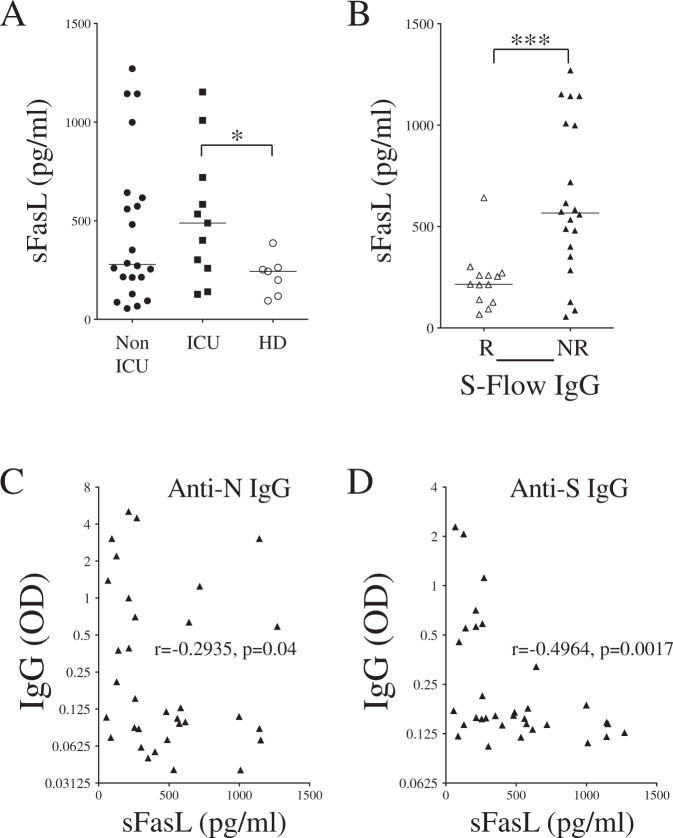
Plasma sFasL and IgG response. **A** Dot plots show the levels of soluble FasL (sFasL) in the plasma of non-ICU and ICU individuals compared to healthy donors (HD). **B** Levels of sFasL in either S-flow responders (R, blank symbols) or nonresponders (NR, close symbols). **C**, **D** Correlations between sFasL levels and anti-N (**C**) and anti-S1 **D** IgG responses. Each dot represents one individual. Statistical analysis was performed using a Mann–Whitney *U* test (*p < 0.05 and ***p < 0.001). Correlations were assessed using the Spearman test (r and p are indicated).

Because higher levels of sFasL in the plasma of hospitalized COVID-19 patients correlate with the extent of CD4 T-cell apoptosis [27], we assessed the levels of sFasL in S-flow responders and nonresponders (not all patients were tested due to the limited available samples). The levels of sFasL were higher in ICU patients (Fig. 10A) and, even more strikingly, in S-Flow nonresponders than in responders (Fig. 10B). We also plotted the IgG responses against N and S1 antigens against sFasL concentration. We observed a negative correlation, indicating that in individuals with high levels of sFasL, the humoral response against SARS-CoV-2 was significantly impaired (Fig. 10C, D).

Altogether, these results demonstrate that individuals with high levels of CD4 T-cell apoptosis and sFasL have a poor ability to build up an effective humoral response.

## Discussion

Our results highlight the fact that the humoral response against S1 is delayed, with a low level of AI (<0.3) in the more severe forms, in which CXCL10 levels and age are high. This contrasts with convalescent individuals in whom specific IgG responses directed against N and S persist for 6 months after infection and demonstrate IgG maturation as indicated by the high level of AI (>0.7). We also demonstrated the importance of analyzing anti-N IgA as an indicator of recent infection. Finally, our study links CD4 T-cell death and sFasL levels during acute SARS-CoV-2 infection with an impaired humoral response. This correlates with the hypothesis that early T-cell immunodeficiency might contribute to the pathogenesis of COVID-19.

There are many reports on serological studies of COVID-19 patients with acute disease (reviewed in ref. [40]). These studies show differences in the substrate (recombinant viral protein/peptides), antibodies tested, and technologies used to evaluate antibodies. However, few differentiate antibody isotypes with high specificity. In our experiment, highly specific secondary antibodies directed against the heavy chain (gamma, alpha, and mu) of these Igs were used to more accurately monitor the humoral response directed against SARS-CoV-2. Thus, ~50% of COVID-19 patients had developed specific IgM directed against N, M, and S1 antigens on the day of hospitalization. However, in convalescent patients, the IgM response was lost upon recovery. This shortness of IgM response is consistent with previous reports showing that IgM peaked in the first weeks after symptom onset and declined thereafter [41]. The N antigen triggers the fastest and strongest humoral response in COVID-19 patients. Thus, within 2 weeks after symptom onset, over 70% of individuals developed anti-IgA against the N protein. This response is independent of patients’ gender and less impacted by age. This high level of IgA in SARS-CoV-2 is consistent with the fact that IgA is the most abundant isotype antibody in the mucosa [42], where it provides first-line immune defense against commensal bacteria, inhaled particles, or pulmonary viral infections. Interestingly, previous reports on SARS-CoV-1 infection indicated that the N protein could activate SMAD3 [43], enhancing transforming growth factor-β (TGFβ) cell signaling, a cytokine required for IgA CSR [44, 45]. Furthermore, IgA dominates in the absence of T helper cells (Tfh) [5], which is essential for B-cell maturation, germinal center (GC) formation, and Ig affinity maturation [46, 47]. A precise analysis of the balance of IgA and IgG against N and S may be beneficial for determining the time of infection. Thus, IgA detection against SARS-CoV-2 might be an early biomarker for clinics as described for other infectious diseases such as *Toxoplasma gondii* [48, 49].

Our results are consistent with and may help to explain previous reports indicating a delay in the emergence of neutralizing antibodies against SARS-CoV-2 in patients who developed the severe disease when compared to milder forms, as well as an attenuated antiviral IgG response in nonsurvivors. Bolouri et al. [11] reported that patients with severe disease presented lower levels of memory B cells over the course of hospitalization whereas, in moderate illness, memory B cells increased more robustly over time throughout hospitalization. In convalescent patients displaying specific anti-S IgG response, specific Tfh increase [13, 50, 51]. Importantly, early depletion of T cells in the lymphoid tissues was associated with defective GCs in the COVID-19 patients who died [26]. Thus, for lymphocyte loss, two indirect mechanisms have been suggested: one mediated by syncytia formation [52] and one related to T-cell apoptosis [27]. Our observation that anti-S1 antibodies from severe COVID-19 patients display a low AI, and are even absent in patients who do not survive, whereas their concentrations increase in convalescent individuals, is consistent with the hypothesis that early defective T-cell help due to T-cell death may compromise IgG antibody production and lower maturation against the S protein of SARS-CoV-2 during the early phase of infection. The lower levels of S-flow IgG response, indicative of reduced neutralizing [39] and ADCC responses in patients with frequent CD4 T-cell apoptosis, are also consistent with this hypothesis. Because these antibodies are crucial in the context of viral infection, not only in blocking viral infection but also in clearing viral-infected cells through ADCC, this may contribute to the viral dissemination recently shown in patients with severe COVID-19 [53]. Likewise, with Ebola virus infection, premature T-cell death due to apoptosis was associated with a lower B-cell response [54]. These observations suggest that, during COVID-19, a compromised humoral response may be the consequence of defective T-cell help due to excessive CD4 T-cell death.

We also found that the age of patients was a critical parameter since most of the ICU individuals aged over 70 had lower humoral responses. Remarkably, the extent of CXCL10 induced by SARS-CoV-2 infection correlates positively with the age of patients, supporting the observation that older individuals have lower humoral responses and are more prone to severe disease and death. Our results are consistent with previous reports indicating a significant delay in the emergence of neutralizing antibodies against SARS-CoV-2 in patients who developed the severe disease when compared to milder forms [7, 8, 10, 55]. Altogether, these results are in disagreement with early reports indicating a positive correlation between the level of anti-SARS-CoV-2 antibodies [56] or neutralizing antibodies [57–59] and the severity of the disease. Therefore, the discrepancies observed in terms of early humoral responses may reflect important differences in the ages of patients in the cohorts of the different reports, as most of the recent studies analyzed humoral responses in patients with a median age of 50 years (as reviewed in ref. [40]). It is also interesting that we found that the level of CXCL10, characterizing severe COVID-19 disease [37], was positively correlated with T-cell apoptosis, whereas it was negatively correlated with the levels of antibodies produced. In line with our observation, it has previously been shown that respiratory syncytial virus infection was associated with a defective humoral response, which correlated positively with the levels of CXCL10 [60], and that neutralizing CXCL10 improved ARDS [61]. Based on a database of several single-cell RNA reports [62–64], CXCL10 was mostly expressed in fibroblasts, macrophages/monocytes, and endothelial cells. Delorey’s study [62–64] indicates that inflammatory monocytes (CD14^hi^CD16^hi^), which are RNA + for SARS-CoV-2, were the myeloid cells expressing CXCL10 transcripts compared to noninfected cells. However, CXCL10 transcripts were not reported in Type I and Type II alveolar pneumocytes (AT1 and AT2, respectively), which are the main SARS-CoV-2 target cell types. In the past, other reports have demonstrated that epithelial cells and smooth airway muscle cells release CXCL10 following stimulation with IFN-γ [65–67]. This way, interferon-gamma-inducing factor (IGIF), also known as IL-18, is associated with COVID-19 disease [27, 68, 69] and is a potent inducer of FasL [70, 71]. Therefore, we cannot exclude the possibility that IFN-γ may contribute to the production of both CXCL10 and FasL during COVID-19. Moreover, in addition to lymphoid T cells [72, 73], it has been shown that non-lymphoid tissues may express FasL [74] like the airway epithelial cell subtypes [75]. Thus, epithelial cells could be the population producing both factors.

One of the limitations of our study is the lack of viral load measurements, which were not recorded in patients hospitalized during the peak of the pandemic, although the importance of viremia remains controversial [76, 77]. Furthermore, the absence of lymphoid tissues for analyzing the cellular immune response in greater depth is also a limitation. More particularly, the role of Tfh cells in relation to humoral response deserves further exploration. Indeed, like others, we have shown that the quality and quantity of Tfh cells in lymphoid tissues are crucial for the genesis of high-affinity antibodies that may be affected in infectious diseases like HIV [78, 79].

Globally, our results provide advances in knowledge about the specific humoral response during the acute phase of SARS-CoV-2 infection in which (i) specific IgA against the N protein dominate and could be used as a marker to estimate the date of infection, (ii) specific IgG against the S protein is delayed, and their quantity and quality are lower compared to N, and inversely correlated with age, CXCL10 and FasL levels and, finally, and (iii) lower anti-S response is associated with higher levels of CD4 T-cell death. Consequently, preventing CD4 T-cell death might be a strategy for improving humoral response during the acute phase, thereby reducing viral dissemination and COVID-19 pathogenicity and also specific immune memory.

## Methods

### Study design and participants

#### Overview of enrollment

The bioclinical features of patients recruited at Nîmes University Hospital (France) from April 9 to July 16, 2020 are shown in Table 1. PCR-positive SARS-CoV-2-infected individuals were enrolled. Although patients were admitted to the Infectious Diseases Department (non-ICU) for symptoms of dyspnea and/or deterioration in their general condition, patients with severe acute respiratory distress syndrome were hospitalized in Intensive Care Units (ICUs). Age- and sex-matched healthy controls were used as negative controls (age range, 28–95 years). This study was approved by the Ethics Committee of the Île-de-France. All patients had provided written informed consent. The trial was registered under Eudract/IDRCB 2020-A00875-34 and Clinical Trials NCT04351711. Blood samples were collected at a single time point on admission to the hospital. Blood was collected, and plasma supernatant, obtained after centrifugation, was frozen to −80 °C.

We also analyzed sera samples obtained longitudinally at different time points during hospitalization from a separate cohort of 11 adult patients with laboratory-confirmed COVID-19, admitted for treatment from April 7 to May 7, 2020 (designated as the ‘longitudinal’ cohort). Blood from these patients was collected every 72 h, from admission until discharge, according to a protocol from the Hospital de Braga (Portugal), which had been approved by the Clinical Board and Ethics Committee (ref 69/2020). Patients who did not completely follow the protocol, with any evidence of simultaneous bacterial infection, or patients being treated with tocilizumab were excluded from this sample (Table 2). The clinical characteristics of patients analyzed 6 months after infection according to the Hospital de Braga (Portugal) protocol are given in Table 3.

**Table 3:** Clinical characterization of convalescent individuals.

Parameter	*N* (% or range)
Gender, *n* (%)	
Female	17 (47)
Male	19 (53)
Age (years), mean (range)	54 (20–87)
Oxygen support (hospitalized patients), *n* (%)	
No oxygen required	2 (10)
Conventional oxygen	5 (26)
High-flow oxygen	6 (32)
Mechanical ventilation	6 (32)

### Production of M and N antigens of SARS-CoV-2

The full gene coding for the N protein (accession number YP_009724397, amino acids 1–419) was cloned into a pET24d vector using Nco1 and Xho1 restriction enzymes (Eurogentec). The protein was expressed in baffled flasks containing 800 mL of Terrific Broth media (Emelca Bioscience) supplemented with 2.5% glycerol and 30 µg/mL kanamycin. BL21 (DE3) *E. coli* bacteria were transformed with this plasmid. After reaching an OD^600^ of 0.6 absorbance unit, N protein production was induced by adding 0.5 mM IPTG. After four hours at 37 °C and shaking at 130 rpm, the cells from each flask were centrifuged, frozen in liquid nitrogen, and stored at −80 °C. The next day, 90 mL of lysis buffer A (50 mM Tris, 500 mM NaCl and 5% glycerol, pH = 7.5) were added to each pellet. After homogenization at 4 °C, the cells were sonicated for 4 minutes and then centrifuged at 24,500 rpm for 45 min. The supernatant was passed through a 1 mL nickel-NTA resin (GE, 17-5318-02), which was extensively washed with lysis buffer, and the protein was eluted with 5 mL of 200 mM imidazole-containing lysis buffer A. The protein was then diluted 25 times in buffer B (50 mM Hepes, 150 mM NaCl, 10% glycerol, pH = 7) and further purified on a 1 mL Source 15 S (GE, 17-0944-10) cationic exchange chromatography. Protein N was eluted by applying a gradient with buffer C (50 mM Hepes, 1 M NaCl, 10% glycerol, pH = 7). The sample was then concentrated at 2 mL and applied to a preparative gel filtration column (GE, 17-1069-01) equilibrated in buffer D (50 mM Tris, 200 mM NaCl, 10% glycerol, pH = 7.5). The N protein was eluted as an oligomer.

The M sample was designed to express only the exposed loops of the M protein from the SARS-CoV-2 virus surface, namely amino acids 1–19 (M1) and 72–79 (M2) (accession number YP_009724393). The two peptides were linked by a precision protein site (LEVLFQGP), and the corresponding nucleotides were cloned into a pGEX-3T3 plasmid using EcoR1 and Xho1 restriction enzymes to give the pGEX-M-dipeptide plasmid. All plasmids in this study were sequenced by Eurofins and checked for validation. BL21 (DE3) *E. coli* bacteria were transformed with the pGEX-M-dipeptide plasmid, and the GST-M-dipeptide resulting protein was expressed in baffled flasks containing 800 mL of Terrific Broth media supplemented with 2.5% glycerol and 100 µg/mL ampicillin. After growing the cells for 3 h at 37 °C with shaking at 130 rpm, the temperature was reduced to 20 °C for 1 h prior to inducing GST-M-dipeptide production with 0.5 mM IPTG. After 60 h, the cells from each flask were centrifuged and frozen in liquid nitrogen. To purify the protein, 90 mL of lysis buffer E (50 mM Tris, 150 mM NaCl, and 10% glycerol, pH = 8.0) were added to each pellet, and the cells were lysed as described above. The supernatant was passed through a 3 mL glutathione resin (GE, 17-5279-01) which was extensively washed with buffer E. The protein was then eluted with 20 mM reduced glutathione containing buffer E, and adjusted to pH = 8. After concentration, the sample was applied to a preparative gel filtration column equilibrated in buffer D. Proteins were then buffer exchanged three times in buffer D and concentrated to 2 mg/mL prior to Elisa assays.

#### Cell death monitoring

Blood cells (5 × 10^5^ cells per ml) were cultured for 12 h in RPMI 1640 supplemented with 10% FCS (PAA Laboratories, Inc), penicillin/streptomycin (50 U/mL, Life technologies), glutamine (2 mM, Life technologies), sodium pyruvate (1 mM, Life technologies), and HEPES buffer (10 mM, Life technologies) at 37 °C and 5% CO_2_. Cell death was assessed by measuring caspase activity with FAM-FLICA reagents (Bio-Rad). Cells were stained with anti-CD3, -CD4, -CD20-specific antibodies (Becton Dickinson). Samples were analyzed by flow cytometry (Attune NxT, ThermoFisher) and using FlowJo software (Tree Star, Inc.).

#### Quantification of CXCL10 and FasL

The amounts of CXCL10 and soluble FasL in the plasma were quantified by ELISA (R&D system). Plates were read at a reference wavelength of 490 nM.

#### IgM, IgA, and IgG humoral responses

Antibody production was monitored by measuring specific Igs by enzyme-linked immunosorbent assay (ELISA) against proteins M, N, and S1 (Sars-Cov-2 S protein S1 carrier-free BioLegend). NUNC MaxiSorp™ well plates were coated with M, N, or S1 proteins (0.5 µg/ml in Tris/Hcl pH 9.6) overnight. After washing and saturation for 1 h with fetal bovine serum (BSA), plasma was serially diluted and incubated for 90 min. Plates were then washed and incubated with goat anti-Human IgG (Fc specific)-peroxidase (A0170, Millipore Sigma), goat anti-Human IgM (Fc specific)-peroxidase (401905, Millipore Sigma), and goat anti-Human IgA (Fc specific)-peroxidase (SAB3701229, Millipore Sigma) for 45 min. These antibodies were specific to the Fc fragments, not recognizing the kappa and lambda chains of the Ig. Secondary antibodies were titrated to optimize sensitivity. After the various washings, substrate reagent solution (R&D systems) was added and incubated for 30 min. The reactions were stopped using sulfuric acid (1 N). The plate was read on a Thermo Scientific™ Varioskan™ reader at wavelengths of 450 nm and 540 nm.

#### SARS-COV-2 spike avidity assay

NUNC MaxiSorp™ ELISA plates (Invitrogen) were used as before for monitoring the anti-S1 IgG response. Once incubated in the presence of serial dilution of plasma, plates were washed with PBS and then incubated for 30 minutes at 37 °C in the absence (PBS) or presence of different urea solutions (1.5, 3, and 6 M). After washing, specific IgG was detected with goat anti-Human IgG (Fc specific)-peroxidase (A0170, Millipore Sigma) and revealed with substrate reagent solution (R&D systems) and, as described above, the plates were thereafter analyzed with Varioskan™ reader. The avidity index (AI) was calculated as follows: AI% = OD value of urea-treated sample/OD of the untreated sample)*100. Indexes with a value above 50% were considered as high IgG avidity; 31% to 49% were considered intermediate IgG avidity, and values below 30% were considered as low IgG avidity.

#### S-flow assay

293 T cells were transfected with a plasmid encoding the full-length S protein (kindly provided by O. Schwartz) [39] or a control plasmid using Lipofectamine 2000 (Life technologies). Twenty-four hours post-transfection, the cells were detached using PBS-EDTA and transferred into U-bottom 96-well culture plates (200.000 cells/well). Cells were saturated with 10% fetal bovine serum at 4 C for 10 min and incubated with the patients’ sera (1:300 dilution) in PBS containing 0.5% BSA for 30 minutes at 4 °C. Cells were then washed and stained for 30 min at 4 °C using a goat anti-Human IgG (Fc specific)-FITC antibody that specifically recognizes the Fc fragment and not the light chains of the Ig (Sigma). After washing, cells were fixed with 2% PFA and analyzed on an Attune™ Nxt flow cytometer using FlowJo software (Tree Star, Inc.).

### Statistical analyses

Statistics were calculated using GraphPad Prism software. A nonparametric Mann–Whitney test and Student’s *t*-test were used for comparison. *P* values indicate significant differences (*, <0.05; **, <0.01; ***, <0.001; ****, <0.0001). Correlations were assessed using the Spearman test. A chi-squared test (X^2^ test) was used to compare frequency.

## Supplementary information

Supplementary figures

Checklist

## Data Availability

The published article includes all the experimental datasets generated and/or analyzed during the current study and are available from the corresponding author upon reasonable request.
