# Supplementary material for: Low quantity and quality of anti-spike humoral response is linked to CD4 T-cell apoptosis in COVID-19 patients
Source: Cell Death Dis. 2022 Aug 27;13(8):741. doi: 10.1038/s41419-022-05190-0 (PMC9419645; doi:10.1038/s41419-022-05190-0)
Supplement: Supplementary file 1 — Supplementary figures [file 41419_2022_5190_MOESM1_ESM.docx]

**Supplementary figures**

**Figure 1. IgM response.** Plasma was serially diluted and tested for IgM against membrane (M), nucleoprotein (N) and spike (S1) proteins. Each dot represents an individual. The dashed lines represent antibody specificity as defined by two SD (Standard Deviation) at 1/800 and IgM negative for HD (Healthy Donor).

**Figure 2. IgA response.** Plasma was serially diluted and tested for IgA against the membrane (M), the nucleoprotein (N), and spike (S1) proteins. Each dot represents an individual. Dashed lines represent antibody specificity as defined by a two SD (Standard Deviation) at 1/400 and IgA negative for HD (Healthy Donor).

**Figure 3. IgG** **response.** Plasma was serially diluted and tested for IgG against the membrane (M), the nucleoprotein (N), and spike (S1) proteins. Each dot represents an individual. Dashed lines represent antibody specificity as defined by a two SD (Standard Deviation) at 1/400 and IgG negative for HD (Healthy Donor).

**Figure 4. Humoral response in convalescent COVID-19 patients.** Plasma from convalescent individuals after 6 months. Igs were tested against the nucleoprotein (N, filled) and spike (S1, blank) proteins. IgM (circle), IgA (square) and IgG (triangle) were diluted at 1/800, 1/400 and 1/400, respectively. Dashed line indicated limit of specificity. Non-parametric Mann Whitney test was used for comparison. (p values: ****, <0.0001). (B) Avidity index of anti-S1 IgG in convalescent individuals. Plasma were serially diluted in the absence or presence of urea (3M). Each dot represents an individual.

**Figure 5. Humoral** **responses in patients with age.** IgM, IgA and IgG responses against either the (A) N protein and the (B) S1 protein were analyzed in ICU and non-ICU individuals aged over 70 (>70) or under (<70) 70 years old. Values are derived from Figure 1. A non-parametric Mann Whitney test was used for comparison (p values: **, <0.01 and ***,<0.001).

**Figure 6. Relationship between humoral response and CXCL10 levels in ICU and non-ICU**. Data from IgG and IgA are derived from Figure 1 whereas values for CXCL10 (pg/ml) are derived from Figure 3. CXCL10 levels were plotted against IgA anti-N (A) and anti-S (B), and against IgG anti-N (C) and anti-S (D). Each dot represents an individual (ICU, filled symbols and non-ICU, blank symbols). Values of Spearman’s correlation are indicated.
